# Prevalence and Spatial Distribution of *Entamoeba histolytica/dispar* and *Giardia lamblia* among Schoolchildren in Agboville Area (Côte d'Ivoire)

**DOI:** 10.1371/journal.pntd.0000574

**Published:** 2010-01-19

**Authors:** Mamadou Ouattara, Nicaise A. N'Guéssan, Ahoua Yapi, Eliézer K. N'Goran

**Affiliations:** 1 Unité de Formation et de Recherche (UFR), Biosciences Université d'Abidjan-Cocody, Abidjan, Côte d'Ivoire; 2 Centre Suisse de Recherches Scientifiques (CSRS), Abidjan, Côte d'Ivoire; New York University School of Medicine, United States of America

## Abstract

**Background:**

New efforts are being made to improve understanding of the epidemiology of the helminths and intensifying the control efforts against these parasites. In contrast, relatively few studies are being carried out in this direction for the intestinal protozoa. To contribute to a better comprehension of the epidemiology of the intestinal protozoa, prevalence, and spatial distribution of *Entamoeba histolytica/dispar* and *Giardia lamblia*, and their association with drinking water supplies, were determined in the Agboville department in southeast Côte d'Ivoire.

**Methods/Findings:**

Stool samples were taken from more than 1,300 schoolchildren in the third year of primary education (CE1) from 30 primary schools and preserved in SAF (sodium acetate-acetic acid-formalin). The samples were analyzed by formalin-ether concentration. Then, a survey questionnaire addressed to schoolchildren and school directors was used to collect data on water supplies. Prevalence of *E. histolytica*/*dispar* and *G. lamblia* were, respectively, 18.8% and 13.9%. No particular focus zone was observed in the spatial distribution of the two species. Significant negative association was observed between use of tap water and high prevalence of *E. histolytica*/*dispar* infection (OR = 0.83, *p* = 0.01). High prevalence of *G. lamblia* infection was positively associated with use of ponds as the source of drinking water (OR = 1.28, *p* = 0.009).

**Conclusion:**

These two species of pathogenic protozoa are present with substantial prevalence in this area of Côte d'Ivoire. Although their spatial distribution is not focused in any one place, determination of the population segments with the highest levels of infection will help to target the chemotherapeutic fight. To reinforce treatment with chemotherapeutic agents, tap water should be made available in all the localities of this area.

## Author Summary

According to WHO, intestinal amoebiasis caused by *Entamoeba histolytica* is the third principal parasitic disease responsible for mortality in the world. This protozoal parasite infects approximately 180 million individuals throughout the world, among whom 40 to 110 thousand die from it each year. Giardiasis, caused by another protozoan parasite, *Giardia lamblia*, infects approximately 200 million individuals throughout the world, is a frequent cause of diarrhea in children, and can have negative impact on growth and development. Unfortunately, these intestinal protozoa are taken into account in few epidemiologic studies. The investigation we carried out to determine prevalence and spatial distribution of these infections shows the importance of these parasites in the Agboville department in southeast Cote d'Ivoire. Determination of spatial distribution of these parasites will help to focus delivery of chemotherapy in this area. In addition, our description of the relation of sources of drinking water with these parasitic infections will contribute to the development of an integrated treatment program for these parasites in this area of Côte d'Ivoire. This work will help make the population and political powers aware of the importance of these parasites and the need for safe drinking water in all localities of this area.

## Introduction

Although intestinal parasites seem to raise much less interest than do AIDS and tuberculosis, they are a major public health problem in tropical regions [1]. In 2002, WHO estimated the number of people infected by digestive tract parasites at 3.5 billion and the number of people made ill by them at 450 million [2]. Whereas much effort is being made toward a better comprehension of helminth epidemiology [3],[4], relatively few equivalent studies are done on intestinal protozoa. This is surprising, because intestinal amebiasis caused by the protozoan *Entamoeba histolytica* is the third-greatest parasitic disease responsible for death in the world after malaria and schistosomiasis [5],[6]. It affects approximately 180 million people, of whom 40,000 to 110,000 die each year [7]. Giardiasis, caused by *Giardia lamblia*, is a frequent cause of diarrhea [8],[9] that can have a negative impact on growth and development of children [10] and affects approximately 200 million people worldwide [11]. These parasitic diseases are found in all the major regions of Africa [12]–[14] and were reported in Côte d'Ivoire by surveys carried out in the west of the country [15]–[18]. *Giardia* cysts were reported in an investigation on an epidemic of diarrhea that occurred in the village of Offoumpo in Agboville area [19]. Other studies in this area also reported a high prevalence of certain protozoal species such as *E. histolytica* [20]. In the same area, N'Guessan et al. found that the very high rate of blood in feces is associated with intestinal schistosomiasis [21]; these authors thought that blood in feces could also be due to other diseases such as amoebiasis. A parasitological survey should help to establish the existence of amoebiasis and assess the probable contribution of *E. histolytica* to the occurrence of fecal blood in the Agboville area.

Treatment of giardiasis and intestinal amoebiasis relies on derivatives 5-nitro-imidazoles such as the metronidazole, marketed since 1959 [22]. To date, some resistant cases of *G. lamblia* to these products have been reported. Unfortunately, no new drug is under development for specific treatment of intestinal protozoa [22]. In order to reduce or delay development of resistance, certain authors recommend avoidance of mass treatments in favor of targeted treatments and greater effort put into prevention [22],[23]. Collection of epidemiological data is necessary to develop fight effective strategies against these parasites.

The main objective of this study was to estimate the prevalence of intestinal protozoa in the feces of schoolchildren in the Agboville area. The secondary objectives were to establish spatial distribution of *E. histolytica* and *G. lamblia* in this area and to determine the relationship between these parasites and household water sources. The results should facilitate evaluation of the endemic level of these parasites and to know if infection risk is focused in an area or is widely spread, and consequently whether massive or focal measures of parasite control are required.

## Methods

### Study site

The study was carried out in the Agboville area, southeast Côte d'Ivoire (3°55′ and 4°40′ West and 5°35′ and 6°15′ North). The area is rugged and consists of numerous valleys with swamps. It is a forested region and the climate is of equatorial type with two rainy seasons and two dry seasons. Its average annual rainfall is between 1,298 and 1,739 mm with temperature ranging between 25 and 26.6°C [24]. This zone covered by a dense hydrographic network made up of two rivers (Agnéby and Mé). The tributaries and streams are numerous and conduct water to some villages; there are also many isolated rivers. The Agboville area has 103 villages and the population is estimated at 244,865 people, most of whom are farmers. The main crops are cocoa, coffee, and food products as in west of the county. Populations are supplied with water by traditional dug wells, boreholes, taps (which are supplied by wells or public water delivery systems), rivers, and ponds.

### Study population

The study population consisted of schoolchildren. The study presented here used two surveys: first, a comprehensive parasitological survey in all the primary schools of one education inspection in the Agboville area that fulfilled our inclusion criteria (i.e., that they were registered in one of the schools of the Agboville inspection); second, a questionnaire survey to collect data on water sources.

### Ethical considerations

Institutional approval of the study protocol was granted by Abidjan-Cocody University (IRB 09-2003). The study received ethical clearance by the Ministry of Public Health in Côte d'Ivoire. Then we obtained the oral consent of teachers and parents of pupils according to the principles of the Declaration of Helsinki, before beginning the data collection. The consent was oral because the majority of the parents cannot read nor write. Documentation of this oral consent was initialed and dated by the examiner according to data collection forms approved by the IRB. It was also approved by the organization of parents of pupils. Participation of pupils was voluntary. Those who refused to give fecal samples or to answer the questionnaire were simply excluded from the study. At the end of the parasitological survey all schoolchildren were treated without cost with albendazole for soil-transmitted helminth infections and intestinal protozoa.

### Parasitological survey: Stool collection and analysis

The list of all 30 primary schools in the area was provided by the inspector. Then, in the last week of October 2004, he explained the aim and the procedures of the study to the school directors and requested class lists with name, age, and sex of each pupil. Sampling was done from all voluntary schoolchildren of the third grade class of the inspection during the last two weeks of November 2004. After the children were given an explanation of the stool sample process, they received plastic, covered 125 ml transparent tubes into which they placed their samples. Tubes were given labels to identify the sample, then placed in racks and transported to the laboratory of the major diseases of Agboville (the state-run laboratoire des grandes endémies d'Agboville). To preserve the samples, 1 to 1.5 g of stool was placed (by wooden stick) in another tube containing 10 ml of sodium acetate–acetic acid–formalin (SAF) solution carrying the same label as the corresponding tube. Thereafter, tubes were shaken vigorously to mix feces and SAF [25],[26]. SAF tubes were transported to the laboratory in Abidjan, where stool samples were analyzed by formol-ether concentration [27] and examined by microscopy. All the species of intestinal protozoa and helminths observed were recorded. Slides were read semiquantitatively for intestinal protozoa (1+, 2+, 3+ according to parasitic load of microscopic fields) and quantitatively for helminths (eggs were counted systematically).

### Questionnaire survey

Two weeks before the parasitological survey, the questionnaire was distributed in all 30 schools. This questionnaire was used in previous studies in Côte d'Ivoire [28],[29]. It takes into account other aspects but only data on water supply sources were considered for this study. It consists of two parts, one sent to school directors and the other to teachers. The part sent to school directors was filled in by them. The part sent to teachers was used to collect data from the students. The teachers followed the instructions that accompanied the questionnaire and interviewed pupils separately, one after another, in an empty classroom to avoid the influence of others on their responses. Answers to the questions were “O” for “yes”, “N” for “no” and “—“ for “I do not know.” Completed questionnaires were collected during the parasitological investigations.

### Spatial distribution of protozoal pathogens

During the parasitological investigation, geographical coordinates and altitude of each school were recorded with GPS (global positioning system; Magellan 315, Thales Navigation, Santa Clara, California, United States); then information on roads were identified on-site and those on rivers were observed on maps. Data were used to develop geo-referenced files of the Agboville area from existing maps, ArcView (Redlands, California, United States), and MapInfo. The prevalence of parasites (by species) was then incorporated into the digital map.

### Statistical analysis

Data were double entered and validated with EpiInfo 2002 (US Centers for Disease Control and Prevention, Atlanta, Georgia, United States). Two age groups, 6–10 and 11–12 years, were performed. Chi-square (χ^2^) tests were conducted with STATISTICA 6.0 (StatSoft, Data Analysis Software System, Tulsa, Oklahoma, United States), to determine the relationship between parasites and the children's age and sex with a confidence interval (CI) of 95%. The relationship between the prevalence of different species of parasites was evaluated by the Pearson correlation coefficient (*r*) and its significance (*p*-value) by linear regression carried out with STATISTICA 6.0. Associations between parasite prevalence and water supply sources were examined by logistic regression conducted with STATA 9.0 (Stata, College Station, Texas, United States).

## Results

### Prevalence and associations of parasites

**Figure 1:**
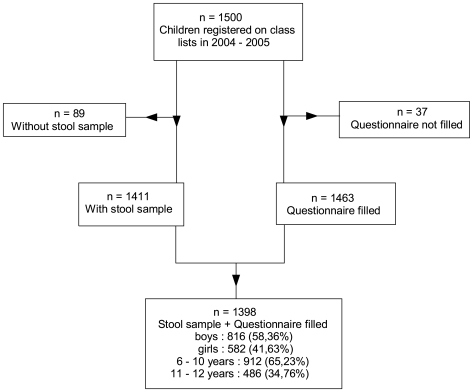
Study profile.

All 30 schools of the Agboville inspection participated in the study. Out of 1,500 schoolchildren who were registered on the class lists, 89 did not provide stool samples (27 were absent during the study and 62 refused to participate) and 37 did not complete the questionnaire (Figure 1). Consequently, 1,398 schoolchildren (93.2%) provided stool samples and answered the questionnaire. Only these were included in the analysis. Eight species of intestinal protozoa, including two pathogenic species, were found in the stool samples. *E. histolytica/dispar* was found in 263 pupils (18.8%) (Table 1) and *G. lamblia* was found in 195 (13.9%). 2.9% of the pupils were infected by both species and 29.7% were infected by at least one of them. In addition to these pathogenic species, six nonpathogenic species were found among the samples: *Entamoeba hartmanni*, *Entamoeba coli*, *Endolimax nana*, *Iodamoeba butschlii*, *Chilomastix mesnili*, and *Blastocystis hominis*. The most common species were *E. nana* and *E. coli*, with respective prevalence of 65.5% and 62.3%. Concerning the prevalence of protozoal infection by age and sex, we found a significant association between the prevalence of *G. lamblia* and sex (χ^2^ = 7.32, df = 1, *p* = 0.006) and between the prevalence of *C. mesnili* and age (χ^2^ = 4.25, df = 1, *p* = 0.037). No other significant association with age and sex were found.

**Table 1:** Number of individuals infected by intestinal protozoa and prevalence in 30 villages of the Agboville area.

Group	Species	Infected Individuals (*n*)	Prevalence (95% CI) (%)	Infected Localities (*n*)
**Pathogenic protozoa**	*Entamoeba histolytica/dispar*	263	18.81 (15.56–22.05)	30
	*Giardia lamblia*	195	13.98 (11.47–16.49)	30
**Nonpathogenic protozoa**	*Entamoeba hartmanni*	36	2.57 (1.17–3.95)	17
	*Entamoeba coli*	871	62.32 (56.27–68.37)	30
	*Endolimax nana*	916	65.52 (59.75–71.33)	30
	*Iodamoeba butschlii*	97	6.92 (5.40–8.44)	29
	*Chilomastix mesnili*	103	7.31 (5.62–9.01)	28
	*Blastocystis hominis*	526	37.60 (33.46–41.73)	30
**Helminths**	*Schistosoma mansoni*	189	13.52 (6.94–20.11)	27
	*Ancylostoma spp*	266	19.02 (14.87–23.17)	29
	*Ascaris lumbricoides*	178	12.73 (8.99–16.51)	27
	*Trichuris trichura*	181	12.94 (7.72–18.15)	30

### Polyparasitism

We found that only 118 (8.4%) students carried no protozoal species. However, among the infected schoolchildren, 326 (23.3%) were infected by one protozoal species and the remaining children (68.2%) had multiple infections. In the multiple-infection group, 2.5% were infected by *E. histolytica/dispar*, *G. lamblia* and *E. coli* and 12.3% by *E. histolytica/dispar*, *E. nana*, and *E. coli*.

### Spatial distribution of *E. histolytica/dispar* and *G. lamblia*

**Figure 2:**
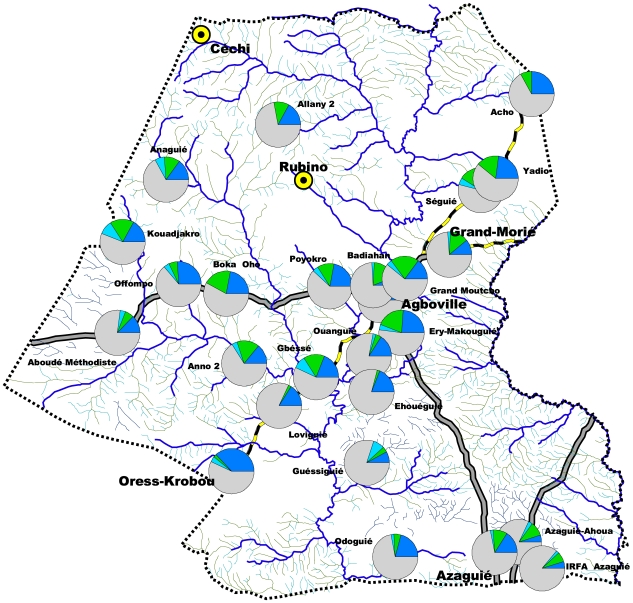
Spatial distribution of *Entamoeba histolytica/dispar* and *Giardia lamblia* in the Agboville area, Côte d'Ivoire.

The five sites studied in the town of Agboville were so close that they merged into a single point on the map. Students in all the study sites in this area were infected by these two parasites (Figure 2). *E. histolytica/dispar* prevalence varied from 4.2% in Agboville town to 40.8% in Oress-Krobou; prevalence exceeded 20% in almost half of the study locations (13 of 30). The villages with the greatest prevalence were distributed throughout the Agboville area. The prevalence of *G. lamblia* ranged from 2.0% in the village of Loviguié to 26.8% at Kouadjakro. Eight localities had prevalence that exceeded 20%: Séguié, Boka Oho, Kouadjakro, Babiahan, Ery-Makouguié, Grand Moutcho, Gbéssé, and Anno. These villages were also distributed throughout the area without focus zone. In addition, Seguié, Boka Oho, Kouadjakro, Ery-Makouguié, Grand Moutcho, and Gbéssé were the most infected localities by both pathogenic species.

### Spatial distribution of *E. histolytica/dispar*, *S. mansoni*, and *Ancylostoma* spp. in localities with a rate of blood in stools over 20%

The spatial distribution of *E. histolytica/dispar* and the rate of blood in stools cannot be superimposed, even if in certain localities prevalence coincides.

Localities where blood in stools is found in more than 20% of the schoolchildren were identified during a study carried out by Guéssan et al. [21]. In our study, prevalence of the three intestinal parasites species suspected to cause blood in stools was evaluated in these localities (Table 2). In Gbéssé, Séguié, and Offompo, only *E. histolytica/dispar* had a prevalence higher than 20%. In these locations, this species is likely the most responsible for the observed blood in stools. In Ery-Makouguié and Oress-Krobou, *E. histolytica/dispar* together with *Ancylostoma* spp. has a prevalence higher than 20%; both species could account for the observed rate of blood in stools. In Odoguié and Yadio, *E. histolytica/dispar* could have slightly contributed to blood in stools, given the higher prevalence of *Ancylostoma* spp. and *S. mansoni*. In all other localities the prevalence of *E. histolytica/dispar* is lower than 20% so this species seems not have contributed to blood in stools there.

**Table 2:** Prevalence of *Entamoeba histolytica/dispar*, *Schistosoma mansoni* and *Ancylostoma* spp. in localities at rate of blood in stools over 20%.

Locality	Prevalence (%)		
	*Schistosoma mansoni*	*Ancylostoma* spp.	*Entamoeba histolytica/dispar*
Azaguié-Ahoua	**48.71**	5.12	7.69
IRFA Azaguié	**45.45**	13.63	6.81
Ery-Makouguié	3.92	**33.33**	**27.45**
Odoguié	**79.06**	16.29	**23.25**
Gbéssé	10.63	19.14	**27.65**
Badiahan	8	3	8
Loviguié	0	12.24	16.32
Oress-Krobou	4.08	**30.61**	**40.81**
Séguié	5.4	18.91	**27.02**
Yadio	**21.62**	**35.13**	**27.02**
Grand Moutcho	4.34	17.39	19.56
Offompo	12	18	**30**
Ouanguié	0	**22**	18
Ehouéguié	7.14	**33.33**	19.04
Guéssiguié 2	11.53	9.61	15.38

Bold indicates prevalence >20%.

### Associations between parasite infections and water sources

Water sources in the Agboville area consist of taps, dug wells, boreholes, rivers, backwaters (shallows area adjacent to or part of a river, where clothing is often laundered), ponds, and reservoirs. Most people supply themselves with water from several sources at the same time. Dug wells are the main source in both towns and villages.

A significant negative association was observed between use of tap water and a high prevalence of *E. histolytica/dispar* (odds ratio OR = 0.84, 95% CI = 0.73–0.96). High prevalence of *G. lamblia* infection had a significant positive association with use of pond water (OR = 1.28, 95% CI = 1.06–1.53). The results show also a significant positive association between high prevalence of non-pathogenic *E. coli* and *E. nana* with, respectively, backwaters and rivers. These two nonpathogenic intestinal protozoa had a significant negative association with use of tap water (Table 3).

**Table 3:** Significant associations between parasites and water supply sources in 30 localities of the Agboville area.

Parasite	Water Source	Odds Ratio (95% CI)	*p*-Value
*Entamoeba histolytica/dispar*	Tap	0.84 (0.73–0.96)	0.01
*Entamoeba coli*	Tap	0.89 (0.82–0.97)	0.008
	Backwater	1.06 (1.00–1.13)	0.035
*Endolimax nana*	Tap	0.89 (0.82–0.97)	0.005
	River	1.09 (1.02–1.17)	0.01
*Giardia lamblia*	Pond	1.28 (1.06–1.53)	0.009

## Discussion

### Prevalence and associations of parasites

The study was carried out among schoolchildren because they were one of age groups the most exposed to intestinal parasites and were generally accessible. Those of third grade primary school (CE1) were chosen because they were the youngest pupils able to answer to questions without difficulty and could be followed over several subsequent years.

The method used for stool analysis, formol-ether concentration [25]–[27], did not allow a distinction between *E. histolytica* and *E. dispar*, so these parasites were indicated by *E. histolytica/dispar*. More specialized methods now exist to distinguish them [30],[31] but remain inaccessible in the majority of developing countries [32]. The prevalence of this parasite complex in our study (18.8%) is identical to that obtained by Heckendorn et al. in 2002 in the town of Agboville [20]. In addition, our extended areas of sampling show that beyond Agboville town, the parasite complex infects people in the wider area (including villages) beyond the town Agboville, and maintains its level of infection in the population. In the Man area, in west Côte d'Ivoire, prevalence of the complex *E. histolytica/dispar* is even lower, with a rate of 11.3% [33]. Distinction between the two species *E. histolytica* and *E. dispar* could led to a weaker prevalence of the pathogenic species [30],[31]. In Agboville town, in an analysis of only microscopically positive samples by PCR, the ratio *E. histolytica* to *E. dispar* was 1∶46 [20]. On the basis of this ratio, prevalence of the pathogenic species (*E. histolytica*) in our study could be about 0.4%. However, studies have shown a significant association between this complex and diarrhea in Nigeria [32], so the high prevalence of the *E. histolytica/dispar* complex as a contributor to illness must nevertheless be considered, even if it is controversial.

Prevalence of *G. lamblia* in Agboville area was 13.9%. This is above other estimates for Côte d'Ivoire: 10.8% in the Man area [18] and 1.4% in Toumodi in central Côte d'Ivoire [34]. The higher prevalence of this parasite in the Agboville area could be due to higher rainfall [24].

Protozoal infection was associated with age and sex for two species. The 6- to 10-year age group was the most infected by *C. mesnili*. This has been observed in the west [17] and in other African countries [1],[35] and is due to the risky behavior and relatively poorer hygiene measures in this age group. *G. lamblia* infection was associated with sex, with girls more highly infected. Where surface water is used for household activities, girls are more vulnerable as indicated by Brelet [36].

### Polyparasitism and spatial distribution

Concerning polyparasitism, our results are comparable to those of Keiser et al. obtained in western Côte d'Ivoire [17]. The observed multiple infections could be explained by the facts that many species of protozoa have the same mode of transmission and that hygiene is poor in these areas. *E. histolytica/dispar* and *G. lamblia* were found in samples from all the localities studied. This cosmopolitan distribution of these parasites has been reported by some authors [7],[31]. Localities of high prevalence are distributed throughout the Agboville area. The even spatial distribution of *E. histolytica/dispar* is identical to that observed in the Man area in western Côte d'Ivoire. In contrast, three focal zones were observed in the spatial distribution of *G. lamblia* in the Man area, contrary to the Agboville area [33]. The even distribution of these parasites in the Agboville area shows that transmission is not related to the physical environment of the area but to the fact of specific parameters of each locality. Eradication efforts should thus take into account the entire area without stratification and emphasize improvements in hygiene conditions. Among the 13 localities with *E. histolytica/dispar* prevalence over 20%, Offompo, Grand Moutcho, Oress Krobou, Yadio, Ery-Makouguié, Odoguié, and Gbéssé also have a fecal blood rate of over 20% [21]. This blood may be due to *S. mansoni* or *Ancylostoma* spp., but *E. histolytica/dispar* is likely to be a cause only in Séguié, Offompo, and Gbéssé. In Azaguié and Agboville, the prevalence of pathogenic protozoan infection is low, as is the blood rate in stool, certainly because these localities benefit from a distribution network of safe drinking water and hygiene conditions are better.

### Water supplies

The socioeconomic status of the populations has not been taken into account in this study. However, recent work has shown that income levels of people influence the distribution of intestinal helminths [37]. Other factors, such as drinking water sources, could play decisive role in the occurrence of these parasites. Therefore, water sources were explored in this study. Negative ratio values between high prevalence of *E. histolytica/dispar*, *E. coli*, and *E. nana* and use of tap water (OR less than 1) show that contamination with these parasites decreases when use of tap water increases. Tap water usually undergoes chemical treatment to remove a number of infectious agents before being distributed to people. These precautions provide relatively good water quality.. Its consumption contributes to the reduction of infection by protozoa. Positive odds ratio (OR greater than 1), obtained between high rates of infection by *G. lamblia*, *E. coli*, and *E. nana* species and use of ponds, backwaters, and rivers as sources of household or drinking water, show that the prevalence of these parasites increase when the use of these sources increases. In Agboville, as in majority of developing countries, hygiene conditions are poor and could support propagation of *G. lamblia* through pond water contamination by human feces. In addition, animals such as rats bathe or drink in ponds and then leave many *Giardia* cysts [38]. These water sources are usually highly polluted, especially in rainy seasons [39], contaminated by rain runoff charged with parasite cysts from animals and human droppings. Consumption of these exposed waters, in an area with high rainfall like Agboville, would be the basis for population-wide parasite infection. As in Offompo village, which has few or no toilets at all [19], other localities studied lack toilets. In villages, when toilets exist, they are not often used and people defecate in the open. This observation was made during a study conducted in a village in Senegal, where 24% of the subjects defecated in the open, despite the existence of toilets [40]. This behavior in the population favors the spread of protozoal cysts.

In order to limit the development of resistant strains of pathogenic intestinal protozoa, some authors recommend focusing preventive efforts and to target chemotherapy [22],[23]. For parasitosis control, spatial distribution is important [41]. In the Agboville area, chemotherapy treatment should target the most infested populations. Sanitation education of the population, especially on the risks of surface water use and precautions to be taken, must accompany this treatment. The importance of providing communities with safe drinking water should also be impressed upon communities and authorities. Implemented together, targeted chemotherapy and provision of safe drinking water will allow better control of these parasites in the study area.

### Conclusion

Intestinal protozoa are common in the Agboville area of Côte d'Ivoire with a high prevalence of the pathogenic species *E. histolytica*/*dispar* and *G. lamblia*. Polyparasitism was highly prevalent in this area. No major focus zone was observed in the spatial distribution of both species. This result shows that control or eradication efforts against these intestinal protozoa must take into account the whole area, with urgent chemotherapy treatments delivered to the most-infected population segments. A significant negative association was observed between infection with *E. histolytica/dispar* and household use of tap water. *G. lamblia* was significantly associated with household use of pond water. Parasites prevalence decreases when tap water is used and increases when surface water is used. This work will help to make populations and political powers aware of the importance of these parasites and the need for safe drinking water in all the localities of this area. It can also contribute to develop an integrated control program against these parasites in this area of Côte d'Ivoire, including prophylactic and chemotherapy measures.

## Supporting Information

Checklist S1 STROBE checklist.
(0.32 MB DOC)
