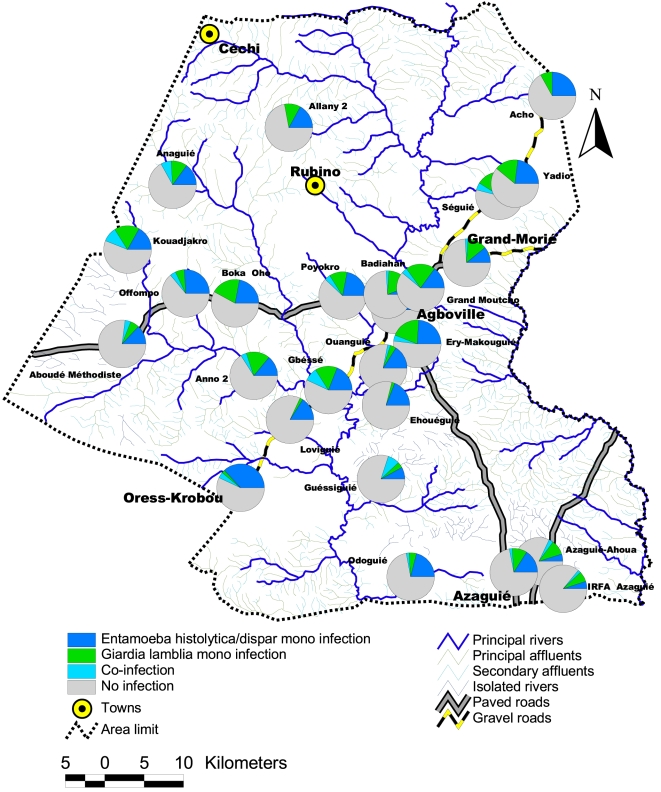# Correction: Prevalence and Spatial Distribution of *Entamoeba histolytica/dispar* and *Giardia lamblia* among Schoolchildren in Agboville Area (Côte d'Ivoire)

**DOI:** 10.1371/annotation/a28fd8eb-b0a9-47f1-935c-7912050dc19b

**Published:** 2010-02-17

**Authors:** Mamadou Ouattara, Nicaise A. N'Guéssan, Ahoua Yapi, Eliézer K. N'Goran

As originally published, Figure 2 lacked its legend. See the corrected figure at 

**Figure:**