# Supplementary figures and images for: Prevalence and Spatial Distribution of Entamoeba histolytica/dispar and Giardia lamblia among Schoolchildren in Agboville Area (Côte d'Ivoire)
Source: PLoS Negl Trop Dis. 2010 Jan 19;4(1):e574. doi: 10.1371/journal.pntd.0000574 (PMC2800181; doi:10.1371/journal.pntd.0000574)

STROBE Statement—Checklist of items


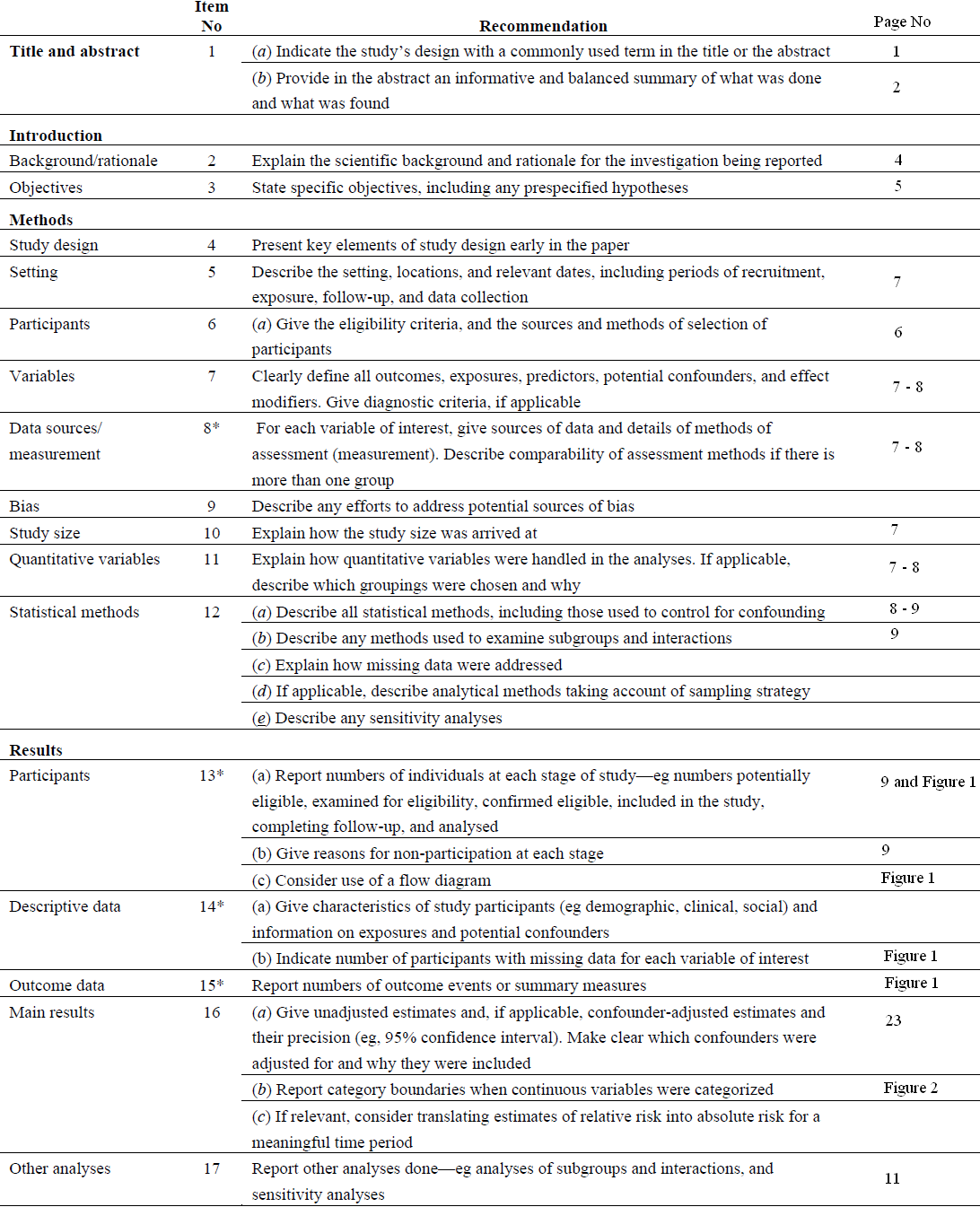


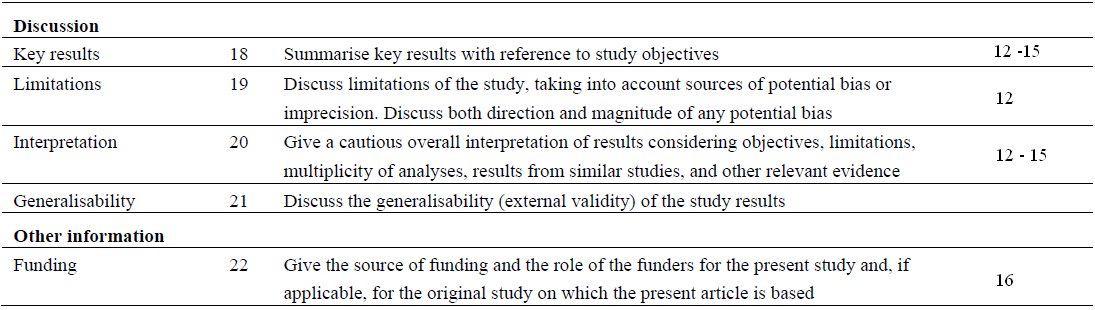

Supplement: Checklist S1 — STROBE checklist. (0.32 MB DOC) [file pntd.0000574.s001.doc]
